# Genetic History of the Remnant Population of the Rare Orchid *Cypripedium calceolus* Based on Plastid and Nuclear rDNA

**DOI:** 10.3390/genes12060940

**Published:** 2021-06-19

**Authors:** Marcin Górniak, Anna Jakubska-Busse, Marek S. Ziętara

**Affiliations:** 1 Department of Molecular Evolution, Faculty of Biology, University of Gdańsk, Wita Stwosza 59, 80-308 Gdańsk, Poland; marcin.gorniak@biol.ug.edu.pl (M.G.), marek.zietara@biol.ug.edu.pl (M.S.Z.); 2 Department of Botany, Faculty of Biological Sciences, University of Wroclaw, Kanonia 6/8, 50-328 Wroclaw, Poland

**Keywords:** cpDNA haplotypes, *Cypripedium*, gen flow, lady’s slipper orchid, Orchidaceae

## Abstract

The lady’s slipper orchid (*Cypripedium calceolus*), which inhabits shady deciduous and mixed forests and meadows, is now threatened with extinction in many European countries, and its natural populations have been dramatically declining in recent years. Knowledge of its evolutionary history, genetic variability, and processes in small populations are therefore crucial for the species’ protection. Nowadays, in south-west Poland, it is only distributed in seven small remnant and isolated populations, which we examined. One nuclear (ITS rDNA) and two plastid (*accD-psa1*, *trnL-F*) markers were analyzed and compared globally in this study. Based on the nuclear marker, the most common ancestor of *C. calceolus* and *Cypripedium shanxiense* existed about 2 million years ago (95% HPD: 5.33–0.44) in Asia. The division of the *C. calceolus* population into the European and Asian lineages indicated by C/T polymorphism started about 0.5 million years ago (95% HPD: 1.8–0.01). The observed variation of plastid DNA, which arose during the Pleistocene glacial–interglacial cycles, is still diffuse in Poland. Its distribution is explained by the result of fragmentation or habitat loss due to human impact on the environment.

## 1. Introduction

*Cypripedium calceolus* (the lady’s slipper orchid or yellow lady’ slipper) is a rare, long-lived, terrestrial, and allogamous orchid species with a horizontal rhizome [1,2,3]. It is a boreal species that occurs in shady deciduous and mixed woodland (rarely in full sunlight) and meadows, predominantly on calcareous soils [3,4]. This orchid is regarded as a species of least concern according to the International Union for Conservation of Nature (IUCN) [5]. Nevertheless, in some countries, including Poland, it is listed in the red list and red books as a threatened species [6] because it is now critically endangered [2,7,8,9]. It has a large distribution area covering most of Eurasia, but in Europe it has suffered a dramatic decline (despite rigorous protection by law) over recent decades, due to over-collection. Most surviving populations today consist of a few plants, and only in a few locations can thousands of plants be found [2,8,10,11,12]. This situation may be mainly explained by climate changes, habitat destruction and alternation, as well as improper forest management [5,9,13].

In Poland, in recent times, extensive habitat loss and improperly implemented protective procedures have reduced the number of *C. calceolus* populations and individuals within populations. Nowadays, about 197 locations have been recorded [14]. Many of them consist of small groups or single individuals (ramets), isolated geographically with restricted effective gene flow. In most of these populations, the number of genets (clonal colony) is currently stable, though not increasing, and only the growth of clones is observed, without effective generative reproduction [8]. Although the population in north-east Poland and the Pomerania region has been well studied [6,12,15], little is still known about the genetic variability within other populations. In Lower Silesia (SW Poland), a gradual disappearance of *C. calceolus* has been observed since the beginning of the 20th century [8,9,16,17,18]. Unfortunately, as a result of deforestation and improperly implemented conservation, a few populations of this rare orchid in Lower Silesia have been destroyed in the last few years, rendering most of the local subpopulations of *C. calceolus* in Lower Silesia unique [9].

Intensive research is currently underway to determine the level of *C. calceolus* genetic variation. Preliminary analysis based on plastid *trnL* intron did not show variation among the Polish populations of *C. calceolus* [12]. However, previous studies conducted by Fay et al. [19] indicated that a maternally inherited plastid genome can serve as a source of information about genetic variation in *C. calceolus* [19]. The most variable marker which differentiated populations was the *accD–psa1* intergenic spacer (IGS), which was also used in this study. Recently, based on the analysis of nuclear DNA using short tandem repeat (STR) variability, Gargiulo et al. [20] indicated that, within the *C. calceolus*, there are four gene pools (A, B, C, D) [20]. Three of them (A, B, D) are found in Europe and Asia, while the C gene pool is characteristic of Far East Russia [20]. Based on plastid DNA variation (*trn*L, *trn*L–F, *psb*A–*trn*H) two haplotypes (H1 and H2) were also identified [15]. Haplotype H1 is characteristic of all four genetic pools (A, B, C, D), while H2 is only found within the genetic pool C in Far East Russia and in *C. shanxiense*. Differences between these haplotypes include three substitutions and seven deletions. In addition, Taniguchi et al. [21] identified a third haplotype typical of individuals from Japan (Hokkaido, Rebun island) and differing in one substitution from the H2 haplotype in the *trn*L-F region. Single-nucleotide polymorphisms were also found in the Internal Transcribed Spacer (ITS) of *C. calceolus.* The T allele occurs in the gene pools A, B, C, D [20] as well as in Korea and Japan (NCBI accession no. KT338681, KT338682, AB176594), while the C allele is characteristic only of the gene pools A, B, and D [20]. The number of substitutions in this region between *C. calceolus* and *C. shanxiense* is five [20].

The aim of this work was to determine the time and place of origin of *C. calceolus* and evaluate the variation between populations of *C. calceolus* in Poland using chloroplast DNA (cpDNA). We also discuss factors which could have affected the distribution of this variability.

## 2. Materials and Methods

### 2.1. Study Species

*C. calceolus* is a rare and endangered orchid species distributed mostly in boreal Eurasia [1,2,3]. It is a tall-growing (height 20–60 (70) cm), perennial geophyte with a horizontal rhizome [2,3]. The species is long-lived, with longevity ranging from 25 to 100 years, depending on clump size [1]. It is a temperate clonal orchid with a relatively high level of vegetative reproduction [1]. The species typically produces one or two, very rarely three, large flowers, in the inflorescence and has a purple-brown perianth and yellow shoe-shaped lip. It does no offer rewards to insects and is mainly pollinated by bees [1]. The overall mean percentage of fruiting plants of this species is mostly low [1]. *C. calceolus* is an allogamous plant [22], and insufficient data about a facultative autogamy in this orchid were reported from the Far East [23]. *C. calceolus* is sensitive to drought; it grows in lightly shaded deciduous and mixed woodlands and in meadows, mostly on calcareous soils, as well as in deciduous and coniferous forests and in bushy hillsides [3,9]. The chromosome number is more or less constant, with most diploid genets having 2n = 20 (22) [3,24,25].

### 2.2. Sampling

The study was conducted in Poland, in south-west (Lower Silesia) and southern (Lesser Poland Province) regions, in 2012–2019. Ten populations of *C. calceolus* were investigated, located in (1) Mt. Młyniec, (2) Mt. Połom, (3) Grudno, (4) Mt. Babilon—population extinct in 2016, (5) a nameless hill next to the Mt. Słupiec—population partially destroyed in 2017–2019, (6) Mt. Wapniarka, (7) Mielnik, (8) Nowa Ligota, (9) Kąty, and (10) Kalina Mała. Locations 1–8 are in Lower Silesia, and those numbered 9–10 are in the Zamosc region and in Lesser Poland. The small sample size is due to small population sizes. Some populations in Lower Silesia consisted of just one or two single ramets in the clone. As *C. calceolus* can show fluctuations in its appearance, we spent several years checking whether new individuals would grow in these populations. Unfortunately, some of these populations still numbered only one or two ramets. In a few cases, we analyzed additional, neighboring ramets, but the results showed that they were all identical, the shoots belonged to one or two genets. The experimental studies and material sampling were done with the permission of the Regional Director for Environmental Protection No. WPN.6400.11.2017.MR.

### 2.3. Molecular Analyses

Plant material was sampled from 42 specimens representing 10 populations of *C. calceolus* from south-western Poland. We also used published data on 3 populations from northern Poland, namely, Las Ostrzycki, Prokowo, and Jezioro Głuche/Bukówki, from the article by Minasiewicz et al. [6], and data from western and north-eastern Poland from an article by Fay et al. [19]—population not identified. The *accD*–*psa1* intergenic spacer (IGS) and internal transcribed spacer (ITS) was used to show genetic variability among populations. In addition, *trnL* intron and *trnL– trnF* intergenic spacer (as one amplicon) were used to distinguish three know haplotypes within *C. calceolus* [20].

### 2.4. Isolation of DNA

Total genomic DNA was extracted from 20 mg silica-dried leaves [26] using a Genomic Mini AX Plant (A&A Biotechnology, Gdynia, Poland). Lysing Matrix A and FastPrep (MP Biomedicals, Irvine, CA, USA) were used to homogenize the samples.

### 2.5. Amplification and Sequencing

The *accD–psa1* region was amplified by using two primers (accD-769F, psaI-75R) referenced in Small et al. [27]. The *trn*L*–trn*L*–trn*F intron and IGS were amplified using *trnl*C and *trn*F primers [28]. Nuclear rDNA (ITS1-5.8S-ITS2) was amplified using 17SE and 26SE primers [29]. Polymerase chain reaction (PCR) amplifications were carried out in a total volume of 25 µL containing of 2.5 µL 10 × buffer, 1 µL 50 mM MgCl_2_, 0.5 µL 10 mM dNTPs, 0.5 µL of 10 µM each of primers, and 1.0 unit of Taq polymerase. The PCR products were purified using a High Pure PCR Product Purification Kit (Roche Diagnostic GmbH, Germany). Cycle sequencing was carried out using a Big Dye Terminator v 3.1 Cycle Sequencing Kit (Applied Biosystems, Inc., ABI, Warrington, Cheshire, UK) with the same primers used for PCR amplification: 2.0 µL of 5× sequencing buffer, 1.0 µL of Big Dye terminator with 1.5 µL of 1 µM primer, 1–4 µL of amplified product (30–90 ng/µL), and H_2_O to a total of 10 µL reaction volume. Both strands were sequenced to assure accuracy in base calling. Finch TV (Geospiza) was used to edit the sequences, and the two complementary strands were assembled by using an AutoAssembler (ABI). All sequences were aligned by eye using a SeaView v. 4 [30]. Sequences were deposited in GenBank.

### 2.6. Haplotype Analyses

Relationships among plastid haplotypes were analyzed with PopArt software (v. 1.7) [31] using median-joining networks based on the variability of the *accD–psa1* region [Supplementary Materials S1. Before the analysis, 14 indels were coded as single characters, so as to treat indels as single events, rather than as multiple independent events [19]. Names of haplotypes refer to haplotypes recognized by Fay et al. [19]. New haplotypes obtained in this study go from H24 to H25.

### 2.7. Molecular Clock Analyses

The ITS rDNA data matrix was used to estimate the tMRCA of *C. calceolus* and formation C/T heterozygosity [Supplementary Materials S2. The data matrix with the exception of *C. calceolus* was taken from Szlachetko et al. [32]. The Bayesian uncorrelated relaxed molecular clock approach implemented in BEAST [33] was used. The Birth Death was chosen as the speciation process. The Akaike Information Criterion in ModelTest v. 3.7 [34] was used to choose the best-fitting evolutionary model (GTR + Γ + I). Two runs were performed in BEAST [33], with 100 million generations each. Log files were analyzed with Tracer v1.5 [35]. All resulting trees were then combined with LogCombiner v1.5.3 [33], with a burn-in of 50%. A maximum credibility tree was then produced using TreeAnnotator v1.5.3 [33]. The value of the time to the most recent common ancestor (tMRCA) of *Cypripedium* was set to that of a normal prior distribution of mean 51 Ma and standard deviation of 4.0 (giving a 95% CI ranging from c. 48–52 Ma), corresponding to the resulting age estimation for *Cypripedium* from the calibration based on the analysis of six chloroplast genes [36]. The original source of the calibration point was based on Ramírez et al. [37].

## 3. Results

### 3.1. ITS Ribotypes/Molecular Clock Analysis

**Figure 1:**
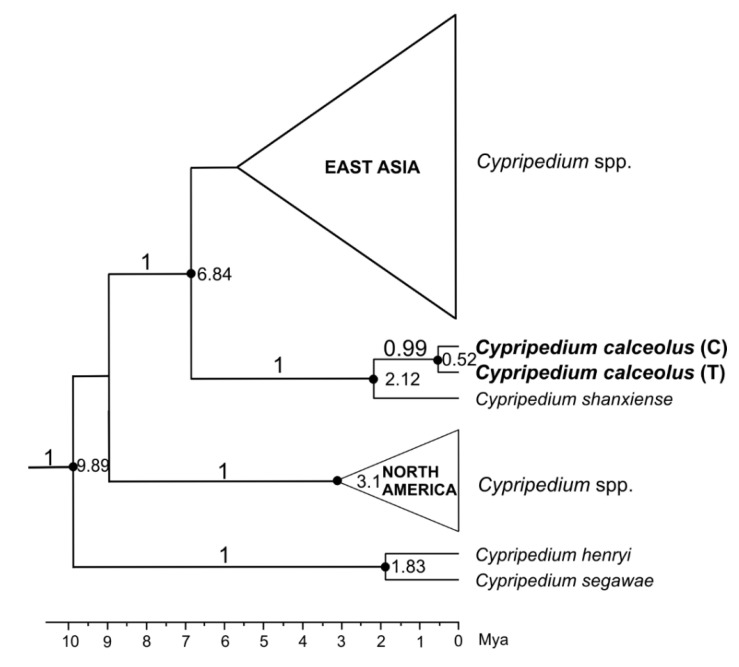
Part of a time-calibrated gene tree of the *Cypripedium* genus (maximum clade credibility trees) resulting from BEAST analysis of the nuclear ITS. Posterior Probability (PP) values > 0.95 are indicated above the branches. The numbers at nodes indicate divergence times in millions of years ago (Mya).

The ITS was sequenced for 42 individuals. One position was variable, detecting two ribotypes (R1—T and R2—C), according to Gargiulo et al. [20]. The intermediate (heterozygous T/C—R3) state was also confirmed. The number of ribotypes was as follows: R1–12, R2–7, R3–23. The time of formation of the T/C mutation within *C. calceolus* was estimated at 0.52 Mya (95% HPD: 1.8–0.01). The time to the most recent common ancestor (tMRCA) for *C. calceolus* and *C. shanxiense* was estimated at 2.12 Mya (95% HPD: 5.33–0.44) (Figure 1).

### 3.2. CpDNA Haplotype Variability

Based on the analysis of the *trn*L intron and *trn*L–*trn*F IGS, one haplotype was detected in all Polish samples. The samples belong to the European lineage (haplotype B) according to Taniguchi et al. [21] and to haplotype 1 according to Gargiulo et al. [20] and as also reported by Brzosko et al. [12]. The aligned matrix of *accD–psa1*, including Polish samples from Fay et al. [19] and Minasiewicz et al. [6], showed 14 indels, ranging in size from 6 to 21 bp, a single base substitution (C/T), and one poly(A) repeat (haplotype data matrix). Eleven haplotypes (Hn) were recognized by combination of the mutations for the 15 different loci, based on the results of the haplotype network analysis (Table 1).

**Figure 2:**
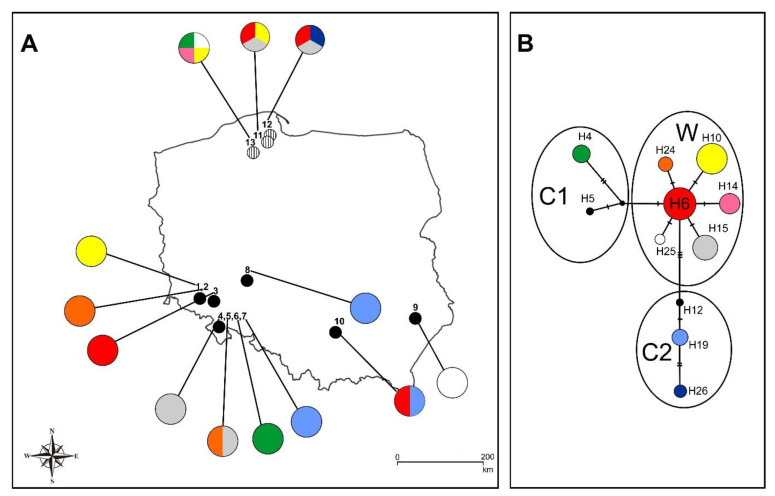
(**A**). Distribution of the investigated *C. calceolus* population in Poland: (1) Mt. Młyniec, (2) Mt. Połom, (3) Grudno, (4) Mt. Babilon, (5) a nameless hill next to Mt. Słupiec, (6) Mt. Wapniarka, (7) Mielnik, (8) Nowa Ligota; (9) Kąty, (10) Kalina Mała, and Gdańsk Pomerania (11,12,13), [6]. Pie charts reflect the frequency of occurrence of each chloroplast haplotype in each population. Haplotype colors correspond to those shown in the network of plastid DNA haplotypes. (**B**). Median-joining network for plastid DNA haplotypes. The haplotypes are indicated by circles, with the size of each circle being proportional to the observed frequency of each haplotype. The number of mutations required to explain transitions among haplotypes is indicated along the lines connecting the haplotypes by cross hatches. W—Western Europe haplogroup, C1 and—C2 Central Europe haplogroup [19].

The haplotypes were grouped into three main lineages (W, C1, C2) (Figure 2B). Lineage W consists of one central (the most common) haplotype, H6, connected by one mutation event with other five haplotypes (H10, H14, H15, H24, H25; see also Table 2 for details of haplotypes distribution). Haplotype 6 was widely distributed across locations in northern (Las Ostrzycki 2_3, Prokowo), south-western (Grudno), and southern (Kalina Mała) Poland. Lineage 2 (C1) consists of two haplotypes (H4 and H5) connected by an ancestral haplotype. The C1 haplogroup is a sister to haplogroup W (Figure 2B). The most diverse haplotypes were found in the lineage C2 at Nowa Ligota, Mielnik, and Kalina Mała (H19), Prokowo (H26), and H12 (sample from Poland, Fay et al. [19]), which are connected by three unique mutation events including one base substitution. The haplotype lineages did not show distinct distribution ranges (Table 2). Most of the haplotypes (with the exception of H14, H19, H24, and H26) are distributed in both northern (one group) and southern (a second one) Poland.

## 4. Discussion

### 4.1. History of Cypripedium calceolus

The *C. calceolus* evolutionary lineage was formed about 2 million years ago in Asia (*C. shanxiense* (a sister species) and other closely related species are found there). This time falls in the Pleistocene period, in which progressive glaciation could have contributed to the geographical isolation of the ancestor of both species. The only observed variation within the ITS region (T/C substitution) emerged in *C. calceolus* about 520,000 years ago (95% HPD: 1.84–0.01 Mya). Based on the presence of state T in other closely related species (*C. shanxiense* and *Cypripedium macranthos*), we consider that this is a plesiomorphic state. The C allele is characteristic of European and Asian populations (Western Russia, Siberia) [20], while the T allele occurs both in Europe and in Siberia and the Russian Far East [20], as well as in Korea and Japan. The emergence of polymorphism within ITS probably occurred after the separation of the ancestral Asian population into two subpopulations, namely, a European one (having both the T and C alleles—gene pool A, B, and D according to Gargiulo et al. [20]) and an Eastern Asia one (having only T alleles—gene pool C according to Gargiulo et al. [20]). A T/C split defines these two subpopulations. It is noteworthy, however, that *C*. *calceolus* individuals from the Russian Far East (gene pool C) have only an H2 haplotype (based on plastid DNA variation, i.e., haplotype A according to Taniguchi et al. [21]), which is also characteristic of *C. shanxiense* [20]. Based on analysis of enzyme systems, Filippov et al. [38] indicated hybridization between *C*. *calceolus* and *C*. *shanxiense* from the eastern part of the range of *C. calceolus*, i.e., the region when these species are sympatric. The question today is whether the diversity of the population from the Russian Far East (gene pool C and H2 haplotype) may be due to the hybridization or to both species sharing an ancient pattern as a consequence of incomplete lineage sorting. Hybridization processes have also been documented in other closely related neotropical species of *Cypripedium* [39].

### 4.2. Plastid Variability of Polish Populations

Based on the analysis of the *accD–psa1* intergenic spacer conducted by Fay et al. [19], the European haplotypes of *C*. *calceolus* form three main groups. The W haplogroup is predominant in Western Europe, and C1 and C2 are predominant in Central Europe [19]. By increasing the number of Polish populations in our analysis, we obtained a similar network structure (Figure 2B). This confirms the findings of the high genetic diversity in the Polish populations of *C. calceolus* found by Fay et al. [19]. Taking into account the slow rate of DNA substitution in the plastid genome and the results obtained on the basis of STR analysis [20], which demonstrated that plants in Italy, France, Germany, Poland, and the United Kingdom belong to the same genetic cluster (gene pool D), it seems that three lineages of plastid DNA (W, C1, C2) were formed before the last glaciation period. Based on the occurrence of the ITS ribotypes—both ribotypes are present in all haplogroups—all three haplogroups were established after the appearance of the C allele (ITS) within *C. calceolus* populations around 520,000 years ago. Over many phases of glaciation, the genetic pool was subjected to differentiation due to isolation in different refugia, in which different lineages could evolve in the absence of gene flow. However, today’s state of genetic variation (nuclear STR) resulted from the last glaciation [20], while the distribution of plastid DNA haplotypes resulted from a redistribution from refugia.

It is noteworthy that identical haplotypes occur in both the North and the South of Poland. The most common haplotype (H6) has been also found in Spain, Sweden, and Switzerland [19]. Previous work, however, showed a lack of gene flow between closely located populations [6] (population no. 11, 12, 13, Figure 2A). Small and isolated populations are often formed from a previously large continuous range as a result of fragmentation or habitat loss due to human impact on the environment [40] and may further suffer from decline of genetic diversity [41,42]. Therefore, the similarity of haplotypes between North and South Poland (a distance of ca. 400 km) can be explained by the fragmentation of the ancestral range during the Holocene and Anthropocene epochs. On the other hand, closely located populations occurring in each of the regions are characterized by a high level of private haplotypes. This situation is due to the extremely small size of the analyzed population. Most of them consist of only a few genets.

## Figures and Tables

**Table 1:** Alignment of plastid haplotypes of *acc*D–*psa1*; − indicates gaps in the alignment; + indicates nucleotides in the alignment.

	Alignment Base Position														
	23–27	28–36	54–59	64–73	121–127	168–175	176–187	395–407	441–446	459–471	489	598–614	541–561	711–717	871–876
Haplotypes															
H4	-	+	+	-	+	+	-	-	+	-	T	-	-	-	+
H5	-	+	+	-	+	+	-	+	+	-	T	-	+	+	+
H6	-	+	+	-	+	+	-	-	+	-	T	-	+	+	-
H10	-	+	+	-	+	-	-	-	+	-	T	-	+	+	-
H12	-	+	+	+	-	+	-	-	+	-	C	-	+	+	-
H14	+	+	+	-	+	+	-	-	+	-	T	-	+	+	-
H15	-	+	+	-	+	+	+	-	+	-	T	-	+	+	-
H19	-	+	+	+	-	+	-	-	+	+	C	-	+	+	-
H24	-	+	-	-	+	+	-	-	+	-	T	-	+	+	-
H25	-	-	+	-	+	+	-	-	+	-	T	-	+	+	-
H26	-	+	+	+	-	+	-	-	-	+	C	_+_	+	+	-

**Table 2:** Plastid DNA variability in populations of *C. calceolus* including the number of haplotypes, sample size, and information about haplotypes localities, compared with data from the literature [6,19]. R1; R2, R3/H1—type of ribotype/haplotype according to Gargiulo et al. [20]. Abbreviation: #Minasiewicz et al. [6]; * Fay et al. [19], AT—Austria, CH—Switzerland, ES—Spain, FR—France, IT—Italy, SE—Sweden, UK—United Kingdom.

Locality		Haplotype (*accd-psa1*)	GenBank Accession No	ITS Ribotype/GenBank Accession No	Haplotype (*trn*L–F)/GenBank Accession No	No. of Genets	*Accd–psa1* Haplotype Localities (Country) *
1	Mt. Młyniec	H10	KF726125	R3/MZ044292	H1/MZ042956	4	FR, ES, CH, UK
2	Mt. Połom	H24	KF726126	R2/MZ044290	H1/MZ042957	3	new haplotype
3	Grudno	H6	KF726127	R3/MZ044293	H1/MZ042958	5	PL, ES, SE, CH
4	Mt. Babilon (population extinct in 2016)	H15	KF726129	R1/MW785560	H1/MZ042959	1	PL
5	A nameless hill next to Mt. Słupiec (south slope)	H24	KF726130	R2/MW785559	H1 MZ042960	4	new haplotype
		H24	KF726130	R3/MZ044294	H1 MZ042960	2	
	A nameless hill next to Mt. Słupiec (north slope)	H15	KF726132	R3/MZ044295	H1/MZ042961	2	PL
6	Mt. Wapniarka	H4	KF726134	R3/MZ044296	H1/MZ042962	5	PL, SE
7	Mielnik	H19	MW883889	R1/MZ044291	H1/MZ042963	6	AT, IT
8	Nowa Ligota	H19	KF726135	R1/MZ044297	H1/MZ042964	1	AT, IT
		H19	KF726135	R3/MW785561	H1/MZ042964	4	
9	Kąty	H25	KF726138	R1/MZ044298	H1/MZ042965	1	new haplotype
10	Kalina Mała	H6	MW883887	R1/MZ044299	H1/MZ042966	2	PL, ES, SE, CH (H6);
		H6	MW883887	R3/MZ044301	H1/MZ042966	1	
		H19	MW883888	R1/MZ044300	H1/MZ042966	1	AT, IT (H19)
11	Las Ostrzycki 1#	H10	KP902524#	-	-	8	FR, ES, CH, UK
	Las Ostrzycki 2_3#	H6	KP902527#	-	-	16	PL, ES, SE, CH (H6)
		H15	KP902525#				PL (H15)
12	Prokowo#	H6	KP902527#	-	-	10	PL, ES, SE, CH (H6);
		H15	KP902525#				PL (H15, H26)
		H26	KP902528#				
13	Jezioro Głuche/Bukówki#	H4	KP902531#	-	-	22	PL, SE (H4)
		H10	KP902530#				FR, UK, ES, CH (H10)
		H14	KP902529#				SE, FR, UK (H14)
		H25	KP902524#				PL (H25)
Poland West, North East, Estonia *		H4	Personal information	-	-	7	PL, SE (H4)
		H5					PL (H5)
		H6					PL, SE, ES, CH (H6)
		H12					PL (H12)
		H15					PL (H15)

## Data Availability

The data presented in this study are available on request from the corresponding author.

## Supplementary Materials

The following are available online at https://www.mdpi.com/article/10.3390/genes12060940/s1, **S1**. Haplotypes data matrix; **S2**. ITS data matrix.
